# Ultrafast Internal Conversion of Aromatic Molecules Studied by Photoelectron Spectroscopy using Sub-20 fs Laser Pulses

**DOI:** 10.3390/molecules19022410

**Published:** 2014-02-21

**Authors:** Toshinori Suzuki

**Affiliations:** 1 Department of Chemistry, Graduate School of Science, Kyoto University, Kyoto 606-8502, Japan; E-Mail: suzuki@kuchem.kyoto-u.ac.jp; Tel.: +81-75-753-3971; Fax: +81-75-753-3974; 2 Molecular Reaction Dynamics Research Team, RIKEN Center for Advanced Photonics, RIKEN, Wako, Saitama 351-0198, Japan; 3 CREST, Japan Science and Technology Agency, Sanbancho, Chiyoda-ku, Tokyo 102-0075, Japan

**Keywords:** photoelectron spectroscopy, femtosecond, laser, filamentation, ultraviolet, imaging, pyrazine, benzene, toluene, internal conversion, ionization

## Abstract

This article describes our recent experimental studies on internal conversion via a conical intersection using photoelectron spectroscopy. Ultrafast *S*_2_(ππ*)–*S*_1_(nπ*) internal conversion in pyrazine is observed in real time using sub-20 fs deep ultraviolet pulses (264 and 198 nm). While the photoelectron kinetic energy distribution does not exhibit a clear signature of internal conversion, the photoelectron angular anisotropy unambiguously reveals the sudden change of electron configuration upon internal conversion. An explanation is presented as to why these two observables have different sensitivities to internal conversion. The 198 nm probe photon energy is insufficient for covering the entire Franck-Condon envelopes upon photoionization from *S*_2_/*S*_1_ to *D*_1_/*D*_0_. A vacuum ultraviolet free electron laser (SCSS) producing 161 nm radiation is employed to solve this problem, while its pulse-to-pulse timing jitter limits the time resolution to about 1 ps. The *S*_2_–*S*_1_ internal conversion is revisited using the sub-20 fs 159 nm pulse created by filamentation four-wave mixing. Conical intersections between *D*_1_(*π*^−1^) and *D*_0_(*n*^−1^) and also between the Rydberg state with a *D*_1_ ion core and that with a *D*_0_ ion core of pyrazine are studied by He(I) photoelectron spectroscopy, pulsed field ionization photoelectron spectroscopy and one-color resonance-enhanced multiphoton ionization spectroscopy. Finally, ultrafast *S*_2_(ππ*)–*S*_1_(ππ*) internal conversion in benzene and toluene are compared with pyrazine.

## 1. Introduction

The quantum mechanical equation of motions of the nuclei and electrons is not exactly solvable, so Born and Oppenheimer approximated the rigorous equation by separating it into the sets of equations of the nuclei and the electrons [1]. Based on the Born-Oppenheimer approximation, a chemical reaction is understood as quantum-mechanical nuclear motion on an adiabatic potential energy surface created by electronic motions. The approximation, however, breaks down at critical nuclear configurations where multiple electronic states have similar energies. Consequently, non-adiabatic transitions occur between different potential energy surfaces in the vicinities of these critical configurations. Non-adiabatic transitions are ubiquitous in the excited state dynamics of polyatomic molecules, because polyatomic molecules have a large number of excited electronic states within a narrow energy range, which cause (avoided) crossings of potential energy surfaces. The most important topographic feature of the surface crossings for non-adiabatic transitions is the conical intersection, which is funnel-shaped in two-dimensional coordinate space but actually is a seam of crossing between multi-dimensional potential energy surfaces [2,3,4,5]. The conical intersection facilitates efficient draining of a nuclear wave packet from an upper to a lower potential energy surface.

Photoelectron spectroscopy, initiated in the late 1950s to early 1960s, [6,7,8] induces emission of an electron from a material using a photon beam (such as UV or X-ray radiation) and measures the kinetic energies of photoelectrons. Time-resolved photoelectron spectroscopy (TRPES) is an advanced form of photoelectron spectroscopy using a pair of laser pulses [9,10,11,12,13,14,15,16,17]. The pump pulse initiates photochemical and/or photophysical processes, and the probe pulse interrogates their time-evolution by photoemission. TRPES requires the laser pulse durations to be shorter than the time scales of the dynamics of interest and the pulse energies sufficiently high to induce ionization within the pulse durations. A highly sensitive electron detection method is also required for TRPES, because the number of photoelectrons generated per an optical pulse-pair must be minimized to avoid electrostatic repulsion between the photoelectrons, which would otherwise alter the electron kinetic energies. Ionization has several important advantages as a probing method of chemical reactions. First of all, the final state is energetically continuous, so that ionization can be induced from any part of the excited state potential energy surfaces as long as the photon energy is sufficient. Secondly, the ionization continuum is degenerate for different symmetries and spin states of photoelectrons, so that ionization is almost always an allowed transition; therefore, the singlet and triplet excited states are equally observed. Additionally, high sensitivity can be obtained by collecting photoelectrons using electromagnetic fields.

Historically, photoelectron spectroscopy was first performed using electrostatic electron energy analyzers [6,7,8]. While these analyzers are still the principal instruments used in high-resolution X-ray photoelectron spectroscopy today, small solid angles and narrow energy windows limit the electron detection efficiencies. With the development of intense pulsed (nanosecond and picosecond) lasers, photoelectron spectroscopy using multi-photon ionization started in the 1980s, which employed time-of-flight (TOF) photoelectron spectrometers. A TOF spectrometer measures flight times of photoelectrons from a sample to a detector, and it enables measurement of the entire photoelectron energy spectrum on a shot-to-shot basis. Furthermore, a magnetic bottle provides an electron collection efficiency of 50% with the TOF analyzer, which enables the application of photoelectron spectroscopy to low-density species such as molecular and metal clusters in supersonic beams [18]. However, a photoelectron angular distribution can hardly be measured with a magnetic bottle, as it utilizes electron cyclotron motions in a magnetic field. Chandler and Houston introduced the two-dimensional (2D) imaging technique in 1987, in which ions emitted from a small target volume in a Wiley-McLaren TOF mass spectrometer are accelerated and projected onto a 2D position-sensitive detector [19]. Eppink and Parker have modified the ion optic electrodes and enabled two-dimensional space focusing of ions to improve the imaging resolution [20]. Their method is now called velocity map imaging, because the arrival position of the ion on the detector plane is proportional to the velocity perpendicular to the flight axis and independent of the ionization point [21]. The performance of the acceleration electrodes (aberration *etc.*) can be further improved by increasing number of electrodes [22,23].

**Figure 1:**
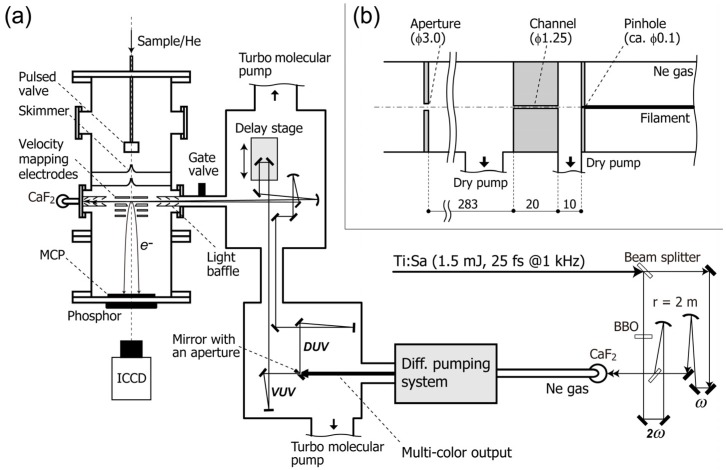
(**a**) Schematic diagram of the experimental setup; (**b**) Schematic drawing of the differential pumping system. All dimensions are given in millimeters. ICCD: image-intensified charge-coupled device camera. MCP: microchannel plate. Reproduced with permission from Ref. [31], copyright (2013) by The Optical Society.

We combined the femtosecond pump-probe method and 2D electron imaging technique to develop time-resolved photoelectron imaging (TRPEI) in 1999 [24,25]. Figure 1 shows a schematic diagram of TRPEI. An ultracold gas of target molecules is created by adiabatic gas expansion into vacuum, and the gas jet is skimmed to create a supersonic molecular beam 2 mm in diameter. The beam is introduced into a photoelectron spectrometer and crossed with the pump and probe laser beams. The pump pulse excites molecules to induce photochemical and/or photophysical processes, and the probe pulse induces photoemission to create an expanding sphere of a photoelectron distribution. The photoelectrons are accelerated in a static electric field and projected onto the 2D detector. The detector consists of microchannel plates, a phosphor screen and a charge-coupled device (CCD) or a complementary-metal-oxide-semiconductor (CMOS) camera, and it records the arrival positions of the photoelectrons on the detector plane. Since both the pump and probe laser polarizations are parallel to each other and to the detector face, the original 3D distribution has axial symmetry around the polarization direction [21,26]. With this symmetry, the 3D distribution can be reconstructed from the projection image. We developed a 2D electron counting apparatus using a CMOS camera and real-time centroiding calculations on a field programmable gate array (FPGA) circuit, which captured electron images at 1 kHz with a uniform sensitivity over the detector area [27].

The photoelectron ejection angle is an important observable in photoelectron spectroscopy. Since the initial ensemble of molecules has an isotropic molecular axis distribution in the absence of an external field, it is an isotropic target for photoionization. Therefore, the anisotropy of the total physical system (molecule + radiation) prior to photoionization is caused by anisotropic electromagnetic field of radiation. This anisotropy is transferred to the PAD after photoionization. For linear polarization of the pump and probe pulses parallel to each other, the photoelectron kinetic energy and angular distribution in [1+1'] photoionization is expressed as follows (the prime means different color):

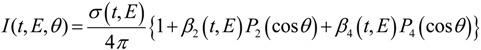
(1)
where *t*, *θ*, and *E* are the pump-probe time delay, the electron ejection angle from the laser polarization direction, and the photoelectron kinetic energy. *P*_n_(x) are the *n*-th order Legendre polynomials. *σ*(*t*,*E*) represents a photoelectron kinetic energy distribution or photoelectron spectrum. *β*_2_(*t*,*E*) and *β*_4_(*t*,*E*) are called anisotropy parameters, and the three scalar quantities *σ*(*t*,*E*), *β*_2_(*t*,*E*) and *β*_4_(*t*,*E*) in Equation (1) are the observables in [1+1'] TRPEI of gaseous samples.

## 2. Generation of Multicolor Sub-20 fs Pulses by Filamentation Four-Wave Mixing

Now laser requirements in TRPEI experiments will be discussed. The most fundamental aromatic molecule benzene has an ionization energy of 9.24 eV, so that one-photon ionization of benzene requires an ionization laser wavelength shorter than 134 nm, and the [1+1'] pump-probe TRPES requires at least one of the pump or probe wavelengths to be shorter than 268 nm. One of the characteristic molecular vibrations of benzene is a totally symmetric ring-breathing mode with a vibrational period of ca. 50 fs. In order to create a spatially-localized wave packet and observe its time-evolution, the pump and probe pulses should be sub-30 fs. While tunable deep UV (DUV) pulses down to ca. 200 nm can be generated using optical parametric amplifiers and non-linear optical crystals, a vacuum UV (VUV) pulse can hardly be generated by these methods. The pulse durations are also generally longer than 30 fs. Thus, we developed a filamentation four-wave mixing method [28,29,30,31] in rare gas to generate sub-20 fs DUV and VUV pulses. Filamentation is a unique propagation scheme of an intense laser pulse through a medium. An intense laser pulse alters the refractive index of the medium (optical Kerr effect), which induces self-focusing of the pulse; however, as the focused laser pulse induces (tunnel-)ionization of the medium, the ionized species create an opposite spatial gradient of the refractive index. Consequently, the laser pulse propagates through the medium as a tightly focused beam for a distance longer than the Rayleigh length [32], which facilitates efficient wavelength conversion in low density gases.

A schematic diagram of our filamentation light source is presented in Figure 1 [31]. A cryogenically cooled Ti:sapphire multipass amplifier delivers ~770 nm pulses (25 fs, 1.5 mJ) at 1 kHz. The fundamental frequency pulse (ω) is split into two beams with a ratio of 3:7. The higher intensity beam is converted to the second harmonic, 2ω, in a *β*-barium borate crystal (*β*-BBO, *θ* = 29°, *t* = 0.3 mm). A dielectric concave mirror (*r* = 2,000 mm) is used to focus the second harmonic into a neon gas cell through a Brewster-angled calcium fluoride (CaF_2_) window (*t* = 1 mm). This concave mirror is also used to separate the second harmonic from the residual fundamental. The fundamental beam is focused into the cell with another dielectric concave mirror (*r* = 2,000 mm). A flat dichroic mirror is used to recombine the fundamental and second harmonic beams. The relative polarization of two beams is parallel to each other. The pulse energies of the fundamental and the second harmonic are 0.43 and 0.37 mJ, respectively. The laser and the optical configuration for the input beams are identical to those employed in our previous study [28] except that the fundamental output from the Ti:sapphire amplifier is 25% lower.

When the ω and 2ω pulses overlap temporally and spatially, a bright filament (plasma column) with a length of ca. 120 mm appears. We use a pinhole (ca. 0.1 mm*ϕ*) instead of an output optical window for the filamentation cell to enable efficient differential pumping while avoiding material dispersion. Behind the pinhole, a narrow channel (1.25 mm in diameter and 20 mm in length) and an aperture (3 mm*ϕ*) are placed for differential pumping, as shown in Figure 1b. The pressure of neon gas in the gas cell is ~3.7 × 10^2^ Torr. The neon gas leaking through the pinhole is evacuated with a dry pump (580 L/min). The pressure in this first differential pumping section is below 3.0 × 10^−1^ Torr. The region between the channel and the aperture is evacuated with another dry pump (500 L/min). The flow rate of neon gas is 0.5 Torr L/s. The laser pulses finally enter a high-vacuum optics chamber that houses an optical delay stage and mirrors. A UV-enhanced aluminum mirror with an aperture of 3 mm diameter spatially separates the central and peripheral parts of the beam. The central part is transmitted through this holed mirror and reflected five times with dielectric mirrors coated specifically for 5ω in order to attenuate other colors. The peripheral part reflected by the holed mirror is used to sample 3ω or 4ω, using dichroic mirrors. The timing of the 5ω pulse is varied using a vacuum-compatible translational stage with 5 nm resolution. The vacuum UV and deep UV pulses are independently focused onto the molecular beam with two Al concave mirrors (*r* =1,000 mm). The intersection angle between the vacuum UV and deep UV pulses is estimated to be ca. 1°. The entire optics chamber is evacuated using two turbo molecular pumps (2 × 300 L/s) to maintain the pressure below 2 × 10^−6^ Torr when operating the filamentation gas cell.

## 3. Two-Dimensional Electron Detector

As mentioned in the introduction, TRPEI is photoelectron spectroscopy that employs an ultrafast pump–probe method and 2D position-sensitive detection of electrons. A 2D position-sensitive detector consists of a microchannel plate (MCP), a phosphor screen, and a digital camera. The MCP is a 70-mm-diameter circular plate that has millions of 10-µm-diameter microchannels over its entire area; the total open area of the microchannels on the MCP surface is ca. 60%. When an electron enters one of the microchannels, an avalanche of secondary electrons occurs and an amplified electron pulse is emitted from the other side of the microchannel. This pulse excites a phosphor screen, visualizing the arrival position of the photoelectrons. The image of the light spot on the phosphor screen is recorded by a CCD sensor or a CMOS image sensor. The acceleration electric field is designed such that the arrival positions of electrons depend only on their velocity vectors and not on the spatial position of ionization. This method thus produces an image of the distribution in *k*-space (*i.e.*, momentum space).

**Figure 2:**
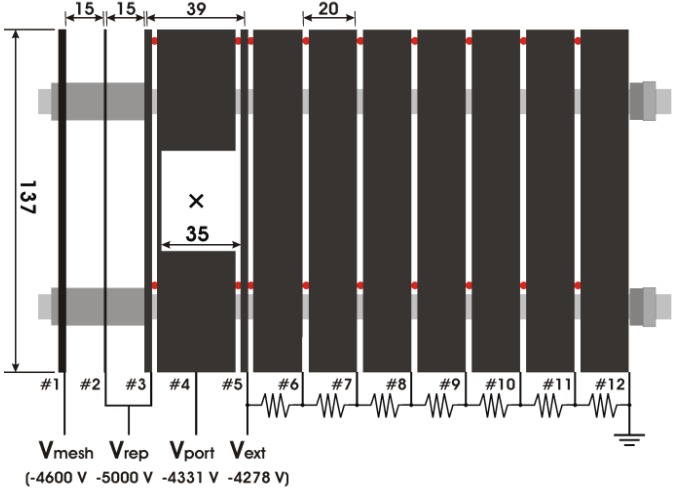
Electrostatic lens system of our imaging spectrometer (all dimensions in millimeters). A molecular beam is introduced from the left. The ionization point is indicated by the cross (×). Reproduced with permission from Ref. [23], copyright (2009) by American Chemical Society.

The design of our electrodes is shown in Figure 2 [23]. A large square hole in electrode 4 allows the propagation of laser beams or He(I) radiation. The ionization point is indicated by a cross (×). We designed electrodes 1–3 to reduce background photoemission due to stray light. Electrode 3 is a repeller plate, but it has a large hole in the center to reduce background photoemission. To flatten the equipotential around the hole in electrode 3, electrode 2 is held at the same voltage as electrode 3. Electrode 1 is used to prevent the ground potential from penetrating the acceleration electric field, while a high-transmission (90%) mesh minimizes its cross section and consequently the background photoemission from electrode 1. Electrode 1 is at a slightly higher potential than electrodes 2 and 3 to prevent photoelectrons emitted from electrode 1 due to stray light being transmitted toward the detector. Electrode 1 has a 6-mm-diameter hole in its center to allow the molecular beam to propagate parallel to the axis of the electrode stack. We computed electron trajectories and found that the velocity resolution improves when even more electrodes are used; however, the velocity resolution saturates in practice. The design shown in Figure 2 provides ∆υ/υ < 0.04% for a focused laser beam.

Even if velocity map imaging electrodes focus the trajectories of electrons with the same velocity, the light spot on the phosphor screen will be considerably larger in diameter than the microchannel pore diameter (10 µm), thereby causing blurring of the photoelectron image. Thus, the center of gravity of each light spot should be calculated to recover the ultimate spatial resolution provided by the velocity map imaging electrodes. The center of gravity of each light spot can be recorded only when the brightness and/or the area of the light spot exceeds a preset threshold for two-dimensional electron counting. This ensures a uniform detection sensitivity over the detector area and highly reliable experimental results [23,27,33]. To perform center-of-gravity calculations and electron counting for each laser shot, the frame rate of the camera must be comparable with or higher than the repetition rate (1 kHz) of the femtosecond laser. We thus constructed a 1-kHz camera using a CMOS image sensor and an FPGA circuit for real-time image processing. A CMOS sensor has a much faster readout than a CCD sensor, although it has a considerably lower sensitivity than a CCD sensor at the present time. We thus use an image intensifier and booster to improve the sensitivity of our camera system; more details are described in the original paper [27].

## 4. Pyrazine: Ultrafast *S*_2_(^1^*B*_2*u*_, ππ*)—*S*_1_(^1^*B*_3*u*_, nπ*) Internal Conversion via Conical Intersection

**Figure 3:**
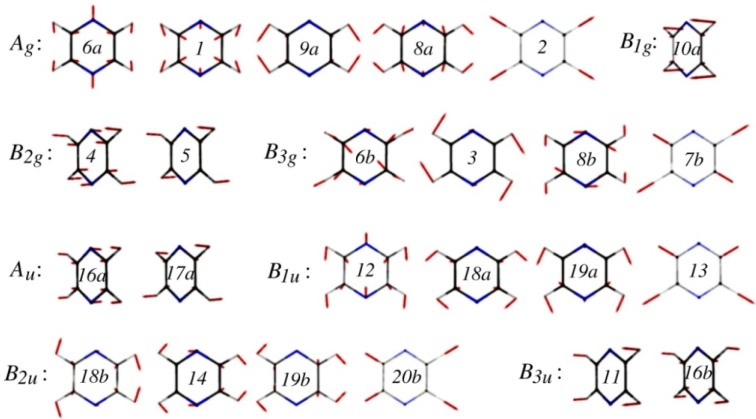
Normal modes of pyrazine calculated at the MP2/aug-cc-pVDZ level. They are labeled using Wilson’s numbering scheme for benzene.

**Figure 4:**
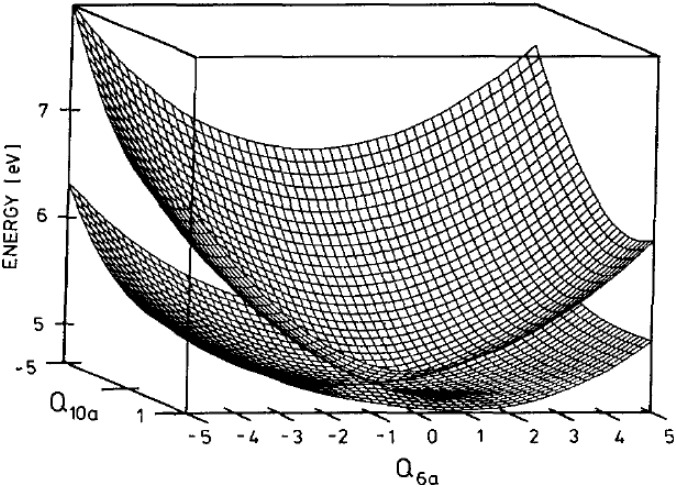
Conical intersection of the *S*_2_ and *S*_1_ adiabatic potential energy surfaces of pyrazine in the two-dimensional space spanned by *Q*_10*a*_ and *Q*_6*a*_. Reproduced with permission from Ref. [19], copyright (1994) by American Institute of Physics.

The *S*_2_(^1^*B*_2*u*_, ππ*)–*S*_1_(^1^*B*_3*u*_, nπ*) internal conversion of pyrazine (C_4_H_4_N_2_, *D*_2*h*_) is one of the best-known examples of ultrafast electronic deactivation via a conical intersection [34]. The topography of this conical intersection has been extensively studied by *ab initio* molecular orbital calculations [35,36,37,38,39,40,41,42,43,44,45,46,47]. Although pyrazine has 24 normal modes (see Figure 3), only a single mode, *Q*_10*a*_(*b*_1*g*_), mediates *S*_2_–*S*_1_ coupling due to the selection rule. Furthermore, only a few totally symmetric (*a_g_*) modes participate in the short-time vibrational dynamics of this system. This reduced dimensionality makes pyrazine a benchmark for theoretical studies of ultrafast internal conversion via a conical intersection. The conical intersection of pyrazine is depicted in Figure 4 for the two-dimensional space of *Q*_10*a*_ and *Q*_6*a*_ [36].

The *S*_2_←*S*_0_ photoabsorption spectrum of pyrazine in the deep ultraviolet region, 230–280 nm, exhibits a broad feature, which implies ultrafast decay of the *S*_2_ state. Despite their broadness, some vibrational structures are discernible in these *S*_2_←*S*_0_ spectra; for example, the progressions of totally symmetric modes, *Q*_1_ and *Q*_6*a*_, are assigned based on comparison of their vibrational frequencies with those in the *S*_0_ state (*ν*_1_ = 1014 cm^−1^ and *ν*_6*a*_ = 596 cm^−1^ for pyrazine-h4) [48]. Several theoretical studies have simulated the spectrum of pyrazine-h4. However, since an absorption spectrum is the Fourier transform of the autocorrelation function of the wave packet prepared by photoexcitation, the absorption spectrum provides limited information about outside the Franck–Condon region. Ultrafast photoelectron spectroscopy enables investigation of nonadiabatic wave packet dynamics over wide regions containing multiple potential energy surfaces.

**Figure 5:**
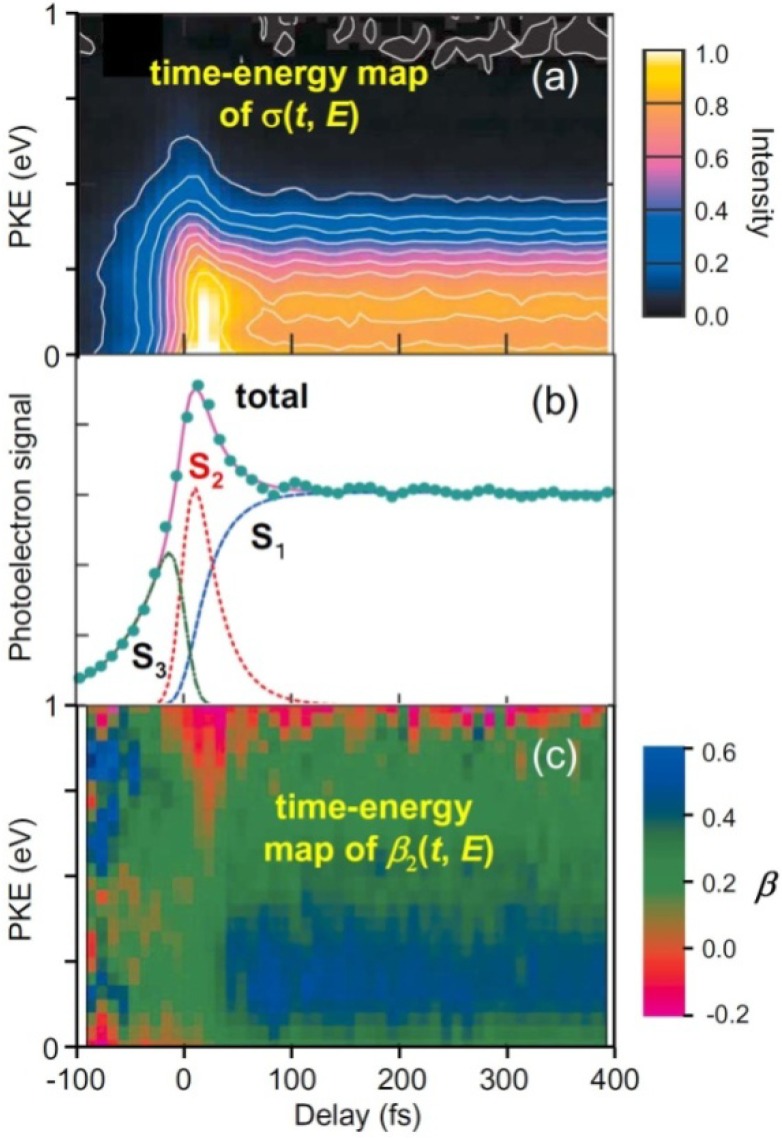
(**a**) Time-evolution of PKED, *σ*(*t*,*E*); (**b**) Temporal profiles of total photoelectron signals in (1+1') TRPEI of pyrazine-h4. The observed data are well explained by three components: single-exponential decay of *S*_2_ (red), corresponding increase in *S*_1_ (blue) at a positive time delay, and single-exponential decay of *S*_3_ (green) at a negative time delay. The fitting result is shown by the solid line; (**c**) Time-evolution of photoelectron angular anisotropy parameter *β*_2_(*t*,*E*).

Figure 5 summarizes our experimental results of TRPEI of pyrazine [49,50,51]. Figure 5b shows the total photoelectron signal as a function of the pump-probe time delay. Since the spectra of our pump and probe pulses overlap the *S*_2_–*S*_0_ and *S*_3_–*S*_0_ bands, respectively, the 264 nm pulse excites ground-state molecules to *S*_2_ and the 198 nm pulse ionizes them for a positive time delay, while for a negative time delay, the roles of the 198 nm and 264 nm pulses are interchanged and molecules are ionized from *S*_3_. The signal at a positive time delay rapidly decays in less than 30 fs and exhibits a plateau; this plateau has a finite lifetime of 22 ps for pyrazine-h4 [25,52]. Furthermore, the plateau region exhibits oscillatory features due to vibrational quantum beats. The Fourier transform of the oscillation (*t* > 50 fs) exhibits a frequency component of 560 ± 40 cm^−1^, which agrees with the vibrational frequency of *Q*_6*a*_ in *S*_1_ (583 cm^−1^). Similarly, pyrazine-d4 exhibits a Fourier component of 550 ± 40 cm^−1^, which provides further support for the assignment to *Q*_6*a*_ (*ν*_6*a*_(*S*_1_) = 564 cm^−1^ for pyrazine-d4) [53]. In the negative time range, the signal diminishes very rapidly within 100 fs (toward the –∞ direction). The observed profile can thus be explained by three components: the decay of optically excited *S*_2_, the corresponding growth of *S*_1_ populated by internal conversion from *S*_2_, and the decay of *S*_3_. By least-squares fitting, the *S*_2_→*S*_1_ internal conversion time constants are estimated to be 23 ± 4 fs for pyrazine-h4 and 20 ± 2 fs for pyrazine-d4. *I*(*t*,*E*), shown in Figure 5a, does not exhibit any marked change on *S*_2_→*S*_1_ internal conversion. This is because photoionization occurs predominantly as *D*_0_(*n*^−1^)←*S*_1_(*n*,π*) and *D*_1_(*π*^−1^)←*S*_2_(*π*,π*), and the energy gaps between *D*_1_ and *D*_0_ (0.88 eV) and between *S*_2_ and *S*_1_ (0.86 eV) are almost the same.

Figure 5c shows a time–energy map of *β*_2_(*t*,*E*). The positive (blue–green) and negative (red) values correspond to preferential ejection of an electron parallel and perpendicular to the probe laser polarization [see Equation (1)]. The energy dependence of *β*_2_, a colored stripe at each time delay in Figure 5c, is a fingerprint of the electronic character. The time–energy map clearly shows that there are three different components, one at a negative time delay and two at a positive time delay, which agrees with the analysis of *σ*(*t*,*E*). The most distinctive feature is the sudden change in the color at ca. 30 fs, which is attributed to ultrafast *S*_2_→*S*_1_ internal conversion. *β*_2_(*t*,*E*) does not change after 30 fs, indicating that the (*n*,π*) electronic character remains; no restoration of the (*π*,π*) character is identified. This lack of recurrence is possibly related to the photoexcitation energy; we excited pyrazine near the *S*_2_ origin. Consequently, if the vibrational energy flows into various modes in *S*_1_, the wave packet has no chance to return to the Franck–Condon region in *S*_2_. Photoexcitation at a shorter wavelength to reach higher vibronic levels in *S*_2_ may enable restoration of the (*π*,π*) character.

**Figure 6:**
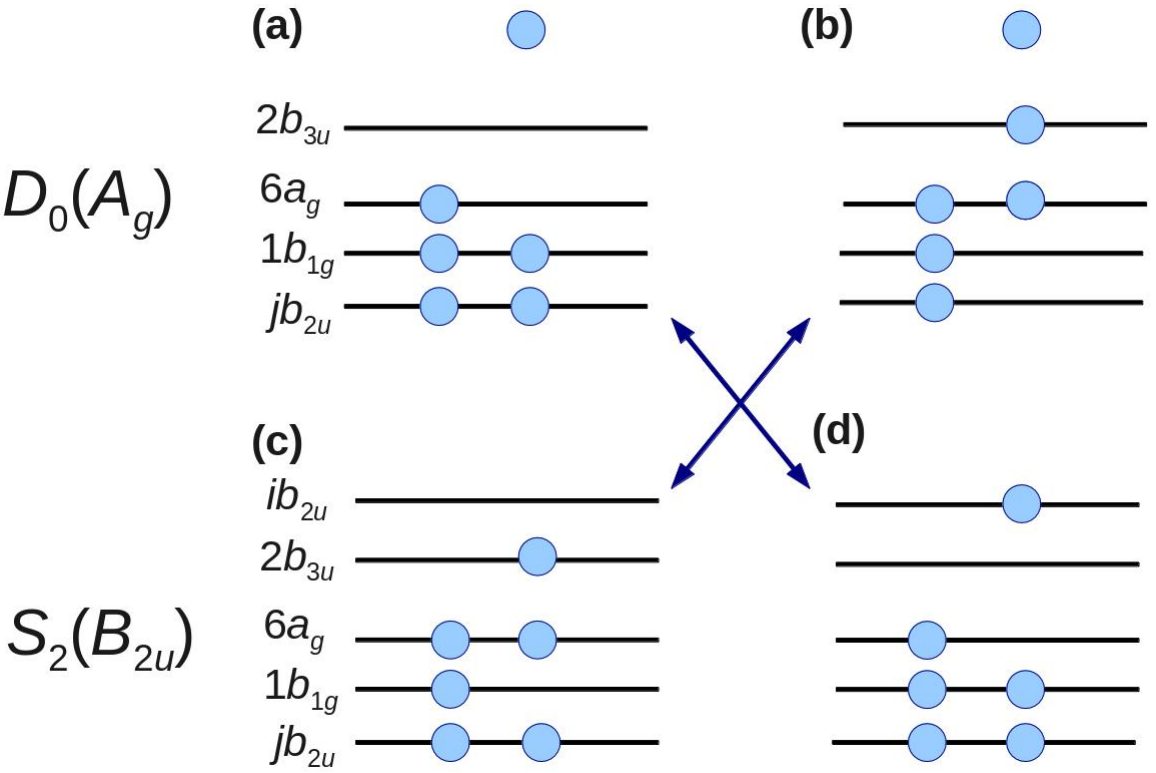
Electronic configurations of *D*_0_ and *S*_2_. The leading configurations of *D*_0_ and *S*_2_ are shown in (**a**,**c**), respectively. By two-electron excitation, configurations (**b**,**d**) are obtained from configurations (**a**,**c**), respectively. Arrows indicate the allowed transition by one-photon ionization. Filled circles represent electrons. Isolated electrons at the top of panels (**a**,**b**) represent photoelectrons.

Note that ionization processes creating PKE < 0.8 eV are mainly *D*_1_←*S*_2_ ionization and *D*_0_←*S*_1_, while PKE > 0.8 eV is created by *D*_0_←*S*_2_ and *D*_0_←*S*_1_. Ionization from *S*_2_ in the latter region is solely due to *D*_0_(*n*^−1^)←*S*_2_(ππ*). This process is forbidden for the main electron configurations of *D*_0_ and *S*_2_; therefore, the occurrence of this ionization process indicates that *D*_0_ and *S*_2_ consist of multiple electron configurations. The *D*_0_ configurations that can be created by one-photon ionization from *S*_2_ are those obtained by removing one electron from an orbital (*ϕ*) of the *S*_2_ configuration, *i.e.*,:

Ψ(*S*_2_) = Ψ(*D*_0_) × *ϕ* (2)

where Ψ(*S*_2_) and Ψ(*D*_0_) respectively denote the electron configurations of *S*_2_ and *D*_0_. Because Ψ(*D*_0_) × *ϕ* should have the same symmetry species as S_2_ [Г(*S*_2_) = Г(*D*_0_) × Г(*ϕ*)], *ϕ* must be the *b*_2*u*_ orbital as given by the direct product *A*_g_(*D*_0_) × *B*_2u_(*S*_2_). However, no *b*_2u_ orbital exists among the outer valence and π* orbitals, which implies that *D*_0_←*S*_2_ cannot be well described by typical valence complete active space self-consistent field (CASSCF) wavefunctions [54]. To make the calculations tractable, we focused on configurations that are doubly excited with respect to the main configuration. Figure 6 shows examples of such configurations. The configurations shown in Figure 6b,d can be obtained by two-electron excitations from the *D*_0_ and *S*_2_ main configurations shown in Figure 6a,c, respectively. Ionization from the configuration in Figure 6d to that in 6a and from the configuration in Figure 6c to 6b is possible. Including these configurations, the first-order configuration interaction calculations account for all one-electron excitations from the complete active space of eight orbitals [*n*, *π*, π* (Figure 6)] From our calculations, we find contributions to the spectral intensity [42] of 47% from 3*b*_2*u*_, 27% from 4*b*_2*u*_, and 26% from all virtual *b*_2*u*_ (*jb*_2*u*_, *j* ≥ 5) [51]. This result clearly demonstrates that the electron correlation cannot be neglected for either *D*_0_ or *S*_2_.

**Figure 7:**
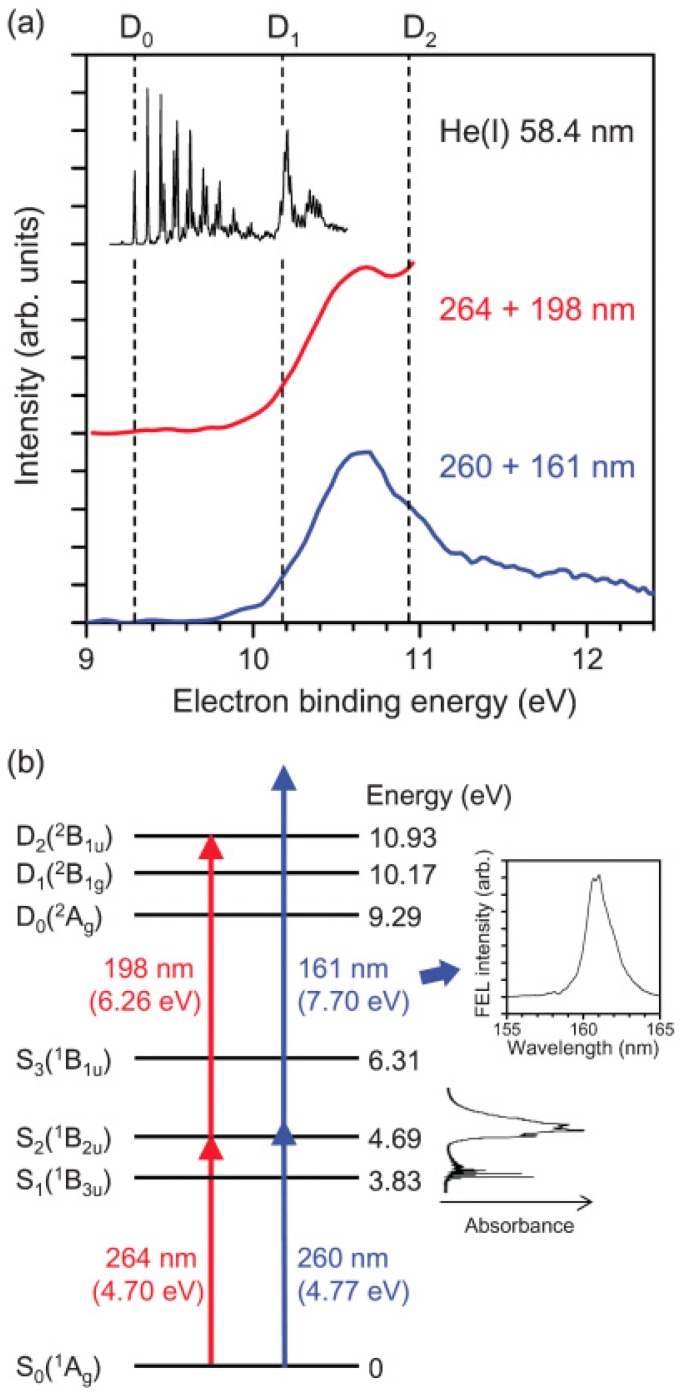
(**a**) PKED in He(I) photoelectron spectroscopy of ground-state pyrazine (black). 264-nm pump and 198-nm probe experiment (red) and 260-nm pump and 161-nm probe (blue); (**b**) Schematic energy diagram of the ionization processes. Insets show UV absorption spectrum of pyrazine vapor at room temperature and time-averaged spectrum of VUV FEL. Reproduced with permission from Ref. [62], copyright (2010) by American Physical Society.

Figure 5 shows that the PKED is rather flat at all times for kinetic energies lower than 0.5 eV. This clearly demonstrates that the Franck–Condon envelopes are not entirely covered for photoionization from *S*_2_ and *S*_1_ due to the probe photon energy being too low. VUV radiation is required to observe the entire envelopes. Femtosecond pulses in the VUV region are currently generated by at least three different methods, namely high harmonic generation using an intense femtosecond laser [55,56,57], free electron lasers [58,59,60], and four-wave mixing [28,29,30,31,61]. We first employed a VUV free electron laser (SCSS: SPring-8 Compact SASE Source) to perform TRPEI experiments in combination with a femtosecond UV laser [62]. Figure 7 compares the photoelectron spectrum measured using the 161 nm probe pulse from SCSS and the 198 nm probe pulse from a filamentation deep UV source in the laboratory [62]. The influence of the pump wavelength can be neglected. The former distribution exhibits the entire Franck–Condon envelope, clearly showing a maximum in the region ca. 1.2 eV above *D*_0_, which is consistent with the vibrational energy of ca. 0.9 eV in *S*_1_. Since *S*_1_ is the (*n*,π*) state and *D*_0_ and *D*_1_ are *n*^−1^ and *π*^−1^ states, the frozen-core approximation predicts ionization occurs from *S*_1_ to *D*_0_, as discussed earlier. Thus, the peak of the photoelectron distribution corresponds to highly vibrationally excited levels in *D*_0_. Nevertheless, since SCSS uses self-amplification of spontaneous emission (SASE) and a thermal cathode, its output pulse intensity, photon energy, and timing inevitably fluctuate. Consequently, the timing jitter between the SCSS and a femtosecond laser is of the order of sub-picoseconds, which did not enable us to observe *S*_2_–*S*_1_ ultrafast internal conversion in pyrazine in real time. As explained above, filamentation four-wave mixing can generate VUV radiation by cascaded four-wave mixing. Therefore, we revisited this problem using a VUV (159 nm) filamentation light source, as described in Section 2.

**Figure 8:**
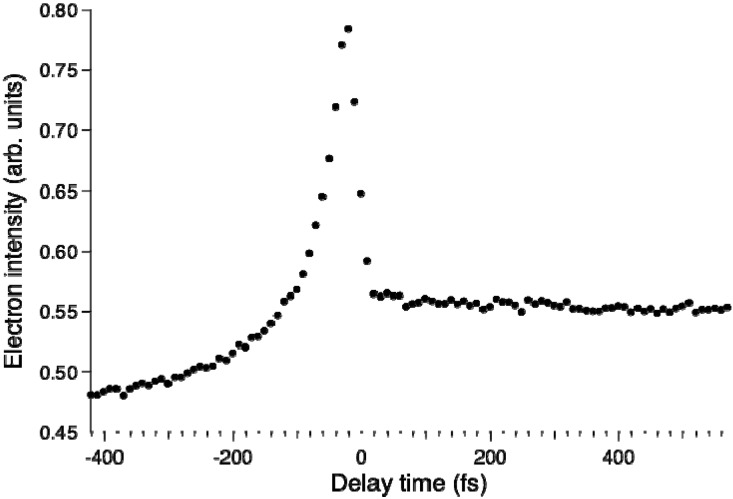
Photoelectron signal time-profile observed for pyrazine using 264 nm pump and 159 nm probe pulses. Negative delay times indicate that the probe pulse precedes the pump pulse.

**Figure 9:**
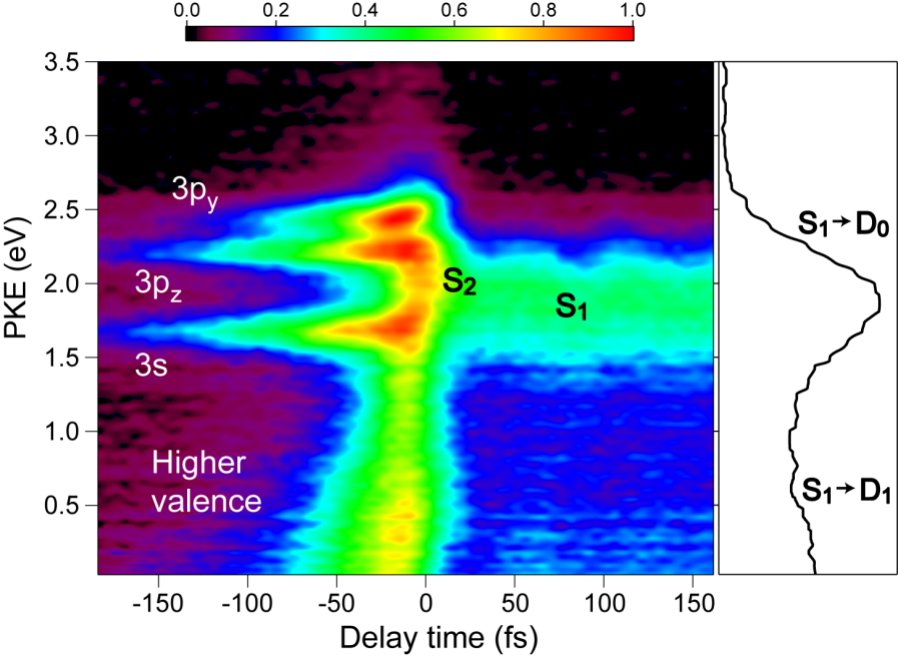
Photoelectron kinetic energy distribution observed for pyrazine using 264 nm pump and 159 nm probe pulses. Negative delay times indicate that the probe pulse precedes the pump pulse. The panel on the right shows the distribution integrated for the time delay of 50–150 fs.

Figure 8 shows the pump–probe time profile of the photoelectron intensity observed using the filamentation light source. The cross-correlation between the 264 and 159 nm pulses is ca.17 fs. The signal intensity is considerably higher in the negative time range where the probe pulse (159 nm) precedes the pump pulse (264 nm). This pulse order excites pyrazine to higher valence states and 3s and 3p Rydberg states and then ionizes from these states. In the positive time range, there is a flat distribution corresponding to the decay of *S*_1_ produced by internal conversion from *S*_2_ pumped by the 264 nm pump pulse. Figure 9 shows the photoelectron kinetic energy distribution. In the negative time range, three Rydberg states of 3s, 3p_y_ and 3p_z_ exhibit sharp horizontal distributions and a valence state exhibits a distribution that diminishes the kinetic energy rapidly and disappears within 100 fs. In the positive time range, the photoionization from *S*_2_ and *S*_1_ are observed, although the signal around *t* = 0 is rather congested due to both the 264 nm pump and 159 nm pump signals. The photoelectron kinetic energy distribution after a pump-probe delay of 100 fs is essentially the same as that obtained using SCSS. More detailed accounts of these results are presented elsewhere.

## 5. Conical Intersections in Cation and Rydberg States of Pyrazine

Similar to *S*_2_(ππ*) and *S*_1_(nπ*), the *D*_1_(*π*^−1^) and *D*_0_(*n*^−1^) potential energy surfaces of pyrazine have a conical intersection [63]. This intersection in the cation raises some interesting questions. First, if ultrafast internal conversion occurs from *D*_1_(*π*^−1^), lifetime broadening should occur in the *D*_1_(*π*^−1^)←*S*_0_ spectrum. The lifetime broadening, however, was not carefully examined in previous studies, because photoelectron spectra were measured for pyrazine vapor at room temperature and the rotational envelopes and vibrational hot bands were not negligible. Second, if ultrafast internal conversion occurs in the cation, similar processes may occur in the Rydberg states because the Rydberg states consist of the same ion core as the cation and a loosely bound Rydberg electron. The question then arises as to whether it is possible to observe the zero kinetic energy photoelectron or pulsed field ionization photoelectron (PFI-PE) spectrum for the *D*_1_(*π*^−1^) state of pyrazine. PFI-PE spectroscopy creates Rydberg states with extremely high principal quantum numbers and high angular momentum quantum numbers and field ionizes them by a pulsed electric field. By scanning the laser wavelength and monitoring the yield of electrons or ions on field ionization, PFI-PE spectroscopy measures an action spectrum that is similar to a conventional photoelectron spectrum.

**Figure 10:**
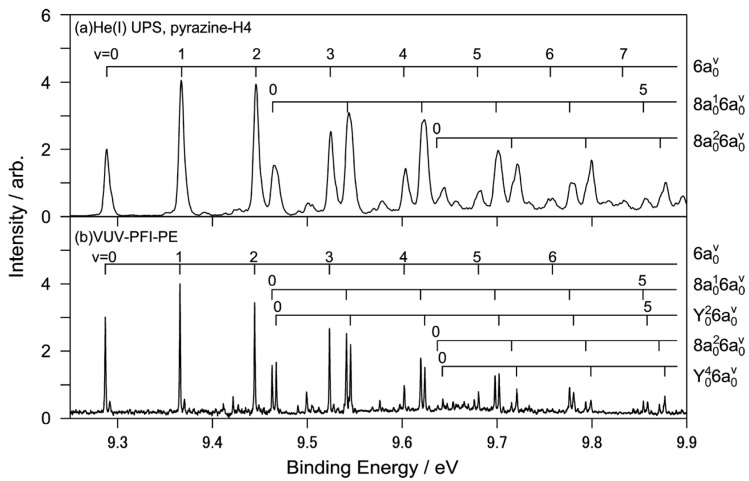
(**a**) Expanded view of He(I) photoelectron spectrum of pyrazine with vibrational assignments; (**b**) VUV-PFI-PE spectra in the *D*_0_(*n*^−1^)←*S*_0_ region. Reproduced with permission from Ref. [64], copyright (2008) by American Chemical Society.

Figure 10a shows the He(I) photoelectron spectrum of jet-cooled pyrazine measured using a He discharge lamp and a hemispherical electron energy analyzer and Figure 10b shows the corresponding region of the PFI-PE spectrum [64]. While both these spectra show one-photon photoionization from the ground electronic state, the former shows direct photoionization, whereas the latter shows resonant excitation to Rydberg states that are energetically almost degenerate with the cation states. Due to the structural change caused by the removal of a valence electron, these spectra exhibit rich vibrational structures that are in remarkable agreement with each other. Close examination reveals that the vibrational temperature is lower in PFI-PE because it employs pulsed expansion of the gas sample to achieve a low vibrational temperature [64]. In contrast, He(I) photoelectron spectroscopy uses a continuous gas jet. The He(I) photoelectron spectrometer has resolutions of 5.5 and 9 meV for pyrazine and fully deuterated pyrazine, respectively, while that of PFI-PE is 1.5 cm^−1^ (0.2 meV).

**Figure 11:**
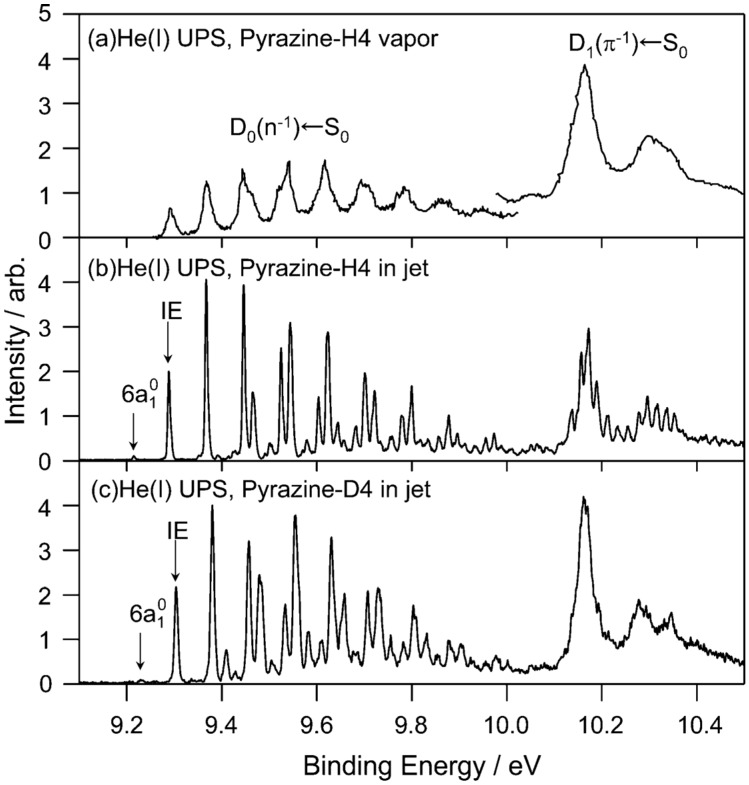
(**a**) He(I) photoelectron spectrum at room temperature reproduced from Ref. [65] with energy recalibration by 83 meV; (**b**) He(I) UPS of pyrazine in a supersonic jet. The spectral resolution is 5.5 meV; (**c**) He(I) photoelectron spectrum of fully deuterated pyrazine in a supersonic jet. The spectral resolution is 9 meV. Reproduced with permission from Ref. [64], copyright (2008) by American Chemical Society.

**Figure 12:**
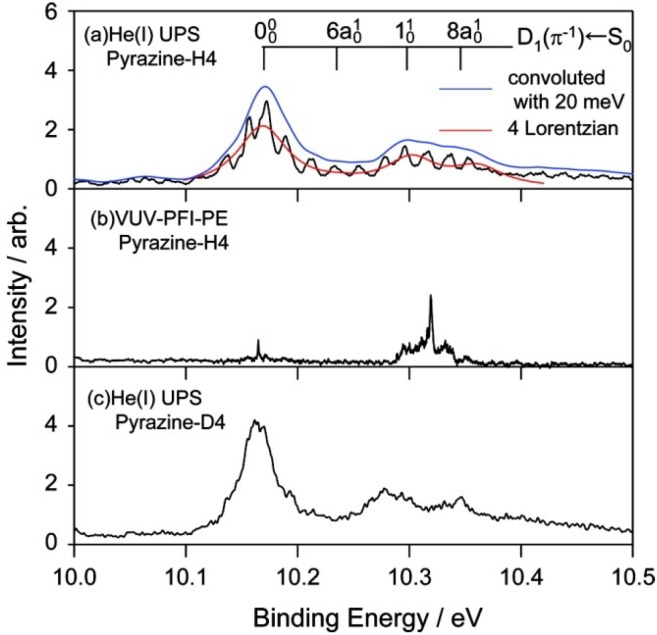
(**a**) Expanded view of the He(I) photoelectron spectrum of jet-cooled pyrazine with vibrational assignments in the *D*_1_(*π*^−1^)←*S*_0_ region. Convolution of the observed spectrum with a virtual instrumental resolution of 20 meV erases structures due to fine splitting. The envelope of the spectral feature is reproduced using four Lorentzian functions for the bands indicated in the figure; (**b**) VUV-PFI-PE spectrum of pyrazine in the *D*_1_(*π*^−1^)←*S*_0_ region; (**c**) He(I) photoelectron spectrum of fully deuterated pyrazine in a supersonic jet. Reproduced with permission from Ref. [64], copyright (2008) by American Chemical Society.

We examine the *D*_1_(*π*^−1^) region in Figure 11, which compares the He(I) photoelectron spectra of pyrazine vapor previously reported [65], jet-cooled pyrazine and fully deuterated pyrazine. Comparison of the photoelectron spectrum of pyrazine vapor (Figure 11a) with our spectrum of a jet-cooled sample (Figure 11b) clearly reveals that the former suffers from instrumental limitations. Our spectra are considerably sharper than the previously obtained spectrum due to supersonic jet cooling of the sample and a higher spectral resolution. The difference in the spectral features in the *D*_1_(*π*^−1^) region is striking: Figure 11a shows only a few broad bands, whereas each of these bands is split into several bands in Figure 11b. Interestingly, the same fine splitting is not observed for fully deuterated pyrazine (Figure 11c). Figure 12 presents expanded views of the *D*_1_(*π*^−1^) region in the three spectra measured for jet-cooled samples. The PFI-PE spectrum in Figure 12b contains sharp bands in the *D*_1_(*π*^−1^) region. However, their features are completely different from those in the He(I) photoelectron spectrum shown in Figure 12a. This result demonstrates that it is difficult to observe a PFI-PE spectrum for the *D*_1_(*π*^−1^) state that undergoes ultrafast internal conversion. We conjecture that the internal conversion mediates couplings with dissociative neutral states and/or ionization continua to induce dissociation into neutral fragments and autoionization. From spectral fitting, the lifetimes of the *D*_1_(*π*^−1^) states of pyrazine and fully deuterated pyrazine are estimated to be 12 and 15 fs, respectively.

**Figure 13:**
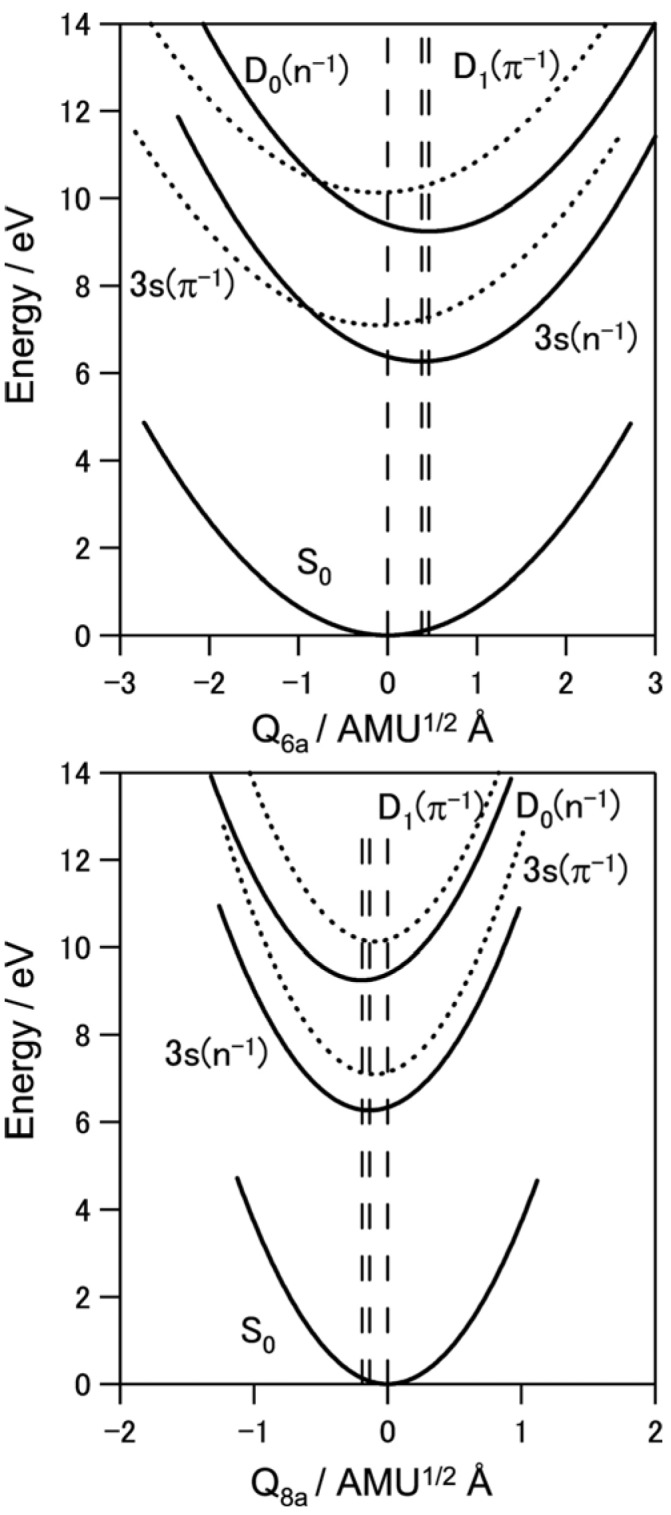
Harmonic potential curves along with 6*a* and 8*a* normal coordinates for pyrazine determined from spectroscopic data. The equilibrium geometries in the 3s(*n*^−1^) and *D*_0_(*n*^−1^) states differ significantly from that of the ground state. The equilibrium geometry of the 3s(*n*^−1^) state differs from that of the *D*_0_(*n*^−1^) state. Reproduced with permission from Ref. [64], copyright (2008) by American Chemical Society.

To estimate the location of the conical intersection point, we analyzed the Franck–Condon factors of the *D*_0_(*n*^−1^) and *D*_1_(*π*^−1^) bands. As seen in Figure 9, *D*_0_(*n*^−1^)←*S*_0_ exhibits vibrational progressions of 6*a* and 8*a* modes. On the other hand, the *D*_1_(*π*^−1^)←*S*_0_ spectrum exhibits a strong 0–0 band, indicating that the equilibrium geometry in *D*_1_ is almost the same as *S*_0_. Franck–Condon analysis provides the magnitudes of the displacements ∆*Q*, but not their signs. Therefore, we determined their signs based on the calculated equilibrium geometry of *D*_0_(*n*^−1^) at the B3LYP/cc-pVTZ level. Figure 13 shows the harmonic potential curves along the 6*a* and 8*a* normal coordinates. Crossings of these potentials are clearly observed for the 6*a* mode. The features of the potential functions thus experimentally determined are in reasonable agreement with the theoretical prediction [63].

**Figure 14:**
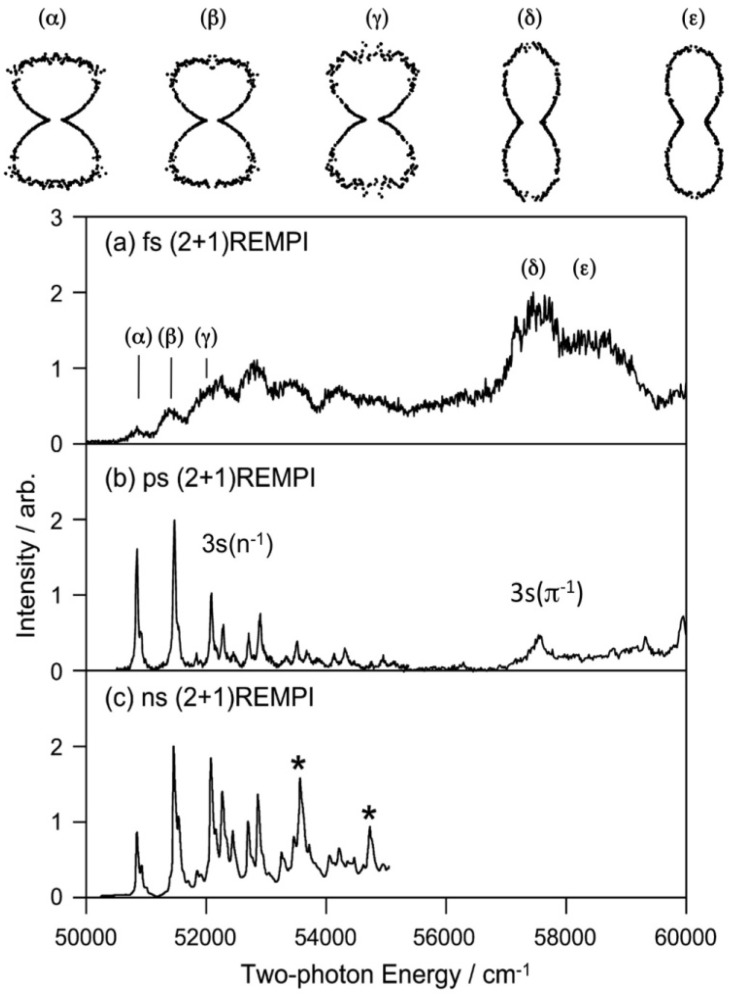
(2+1) REMPI spectra of pyrazine-H4 observed via 3s(*n*^−1^) and 3s (*π*^−1^) Rydberg states with (**a**) a femtosecond laser (150–200 fs); (**b**) a picosecond laser (2.8 ps); and (**c**) a nanosecond laser. The spectrum in (**b**) was measured in the present study by maintaining a constant laser power during the measurement. The spectra in (**a**,**b**) are of molecules in a supersonic jet, while that in (**c**) is of a vapor. The PADs observed for the bands (α)–(ε) are shown as polar plots. The distributions are characteristic of the vibronic bands of 3s(*n*^−1^) and 3s*(π*^−1^) and are useful for their assignments. Reproduced with permission from Ref. [64], copyright (2008) by American Chemical Society, and Ref. [66], copyright (2001) by American Institute of Physics.

The Rydberg states generally have similar potential energy surfaces as those of the cation since the Rydberg electrons with high principal and angular momentum quantum numbers penetrate little into the ion core. For the lowest (3s) Rydberg state, the Rydberg electron penetrates relatively deeply into the core, but it still has quite a similar potential energy surface to that of the cation. Our first study of the 3s Rydberg states of pyrazine was performed using a femtosecond laser, whereas our second study was performed using a picosecond laser. These (2+1) REMPI spectra of pyrazine via 3s Rydberg states are shown in Figure 14 along with the spectrum recorded using a nanosecond laser. All three spectra were recorded by scanning the laser wavelength while monitoring the photoionization signal intensity. The spectrum recorded using a femtosecond laser is very broad due to its wide bandwidth and possible power broadening. This spectrum has not been corrected for variation in the laser intensity.

The spectrum measured with a picosecond laser shown in Figure 14b exhibits a very clear vibrational feature for the 3s(*n*^−1^) Rydberg state and a broad feature for the 3s(*π*^−1^) Rydberg state. Comparison with the *D*_0_(*n*^−1^) photoelectron spectrum reveals that the 3s(*n*^−1^) state mainly differs in that it exhibits lifetime broadening due to interactions with valence electronic states, whereas *D*_0_(*n*^−1^) has no decay; the vibronic band of 3s(*n*^−1^)←*S*_0_ has a width of 15 cm^−1^. Our main interest here is the width of the 0–0 band of 3s(*π*^−1^)←*S*_0_; it is as large as 390 cm^−1^, corresponding to a lifetime of 14 fs. A similar width, 370 cm^−1^, is observed for the 3s(*π*^−1^)←*S*_0_ 0–0 band of deuterated pyrazine. The estimated lifetimes of the 3s(*π*^−1^) Rydberg states are similar to those of *D*_1_(*π*^−1^). Our study clearly demonstrates that ultrafast internal conversion in the ion core also occurs in the Rydberg states.

Figure 14 also shows the PAD measured for each vibronic bands [66]. The PADs observed for 3s(*n*^−1^) and 3s(*π*^−1^) differ greatly, which assists assignment of vibronic bands. PEI is expected to be useful for analyzing complex photoabsorption spectra of higher excited states.

## 6. Internal Conversion of Benzene and Toluene: Comparison with the Case of Pyrazine

Benzene is a prototypical aromatic molecule and considered a benchmark for theoretical and experimental studies of organic compounds. The lifetime of the *S*_1_(^1^*B*_2*u*_) state of benzene is 50–100 ns near the origin, while the *S*_2_(^1^*B*_1*u*_) lifetime is much shorter (<100 fs) owing to ultrafast *S*_2_–*S*_1_ internal conversion. Similar to the case of pyrazine, the ultrashort *S*_2_ lifetime implies that photoexcited benzene easily accesses an *S*_2_/*S*_1_ conical intersection region. In fact, theoretical calculations predicted that the minimum-energy *S*_2_/*S*_1_ conical intersection point (a prefulvenic form) is close, in energy and structure, to the *S*_2_ potential minimum [67,68,69]. The minimum, however, is at a non-planar structure (a boat form) that differs from the planar structure of benzene (*D*_6h_) in *S*_0_ [67,68,69]. Consequently, a photoexcited benzene molecule rapidly deforms from a planar structure in the Franck-Condon region toward a non-planar structure along the steepest descent of the *S*_2_ potential energy surface and undergoes a non-adiabatic transition in the vicinity of the *S*_2_/*S*_1_ seam of crossings. This is a different feature from the pyrazine case in which the Franck-Condon region is close to the minimum energy conical intersection point. The hot *S*_1_ benzene produced by *S*_2_–*S*_1_ internal conversion is further funneled down to *S*_0_ via *S*_1_/*S*_0_ conical intersection in <10 ps [69,70,71].

**Figure 15:**
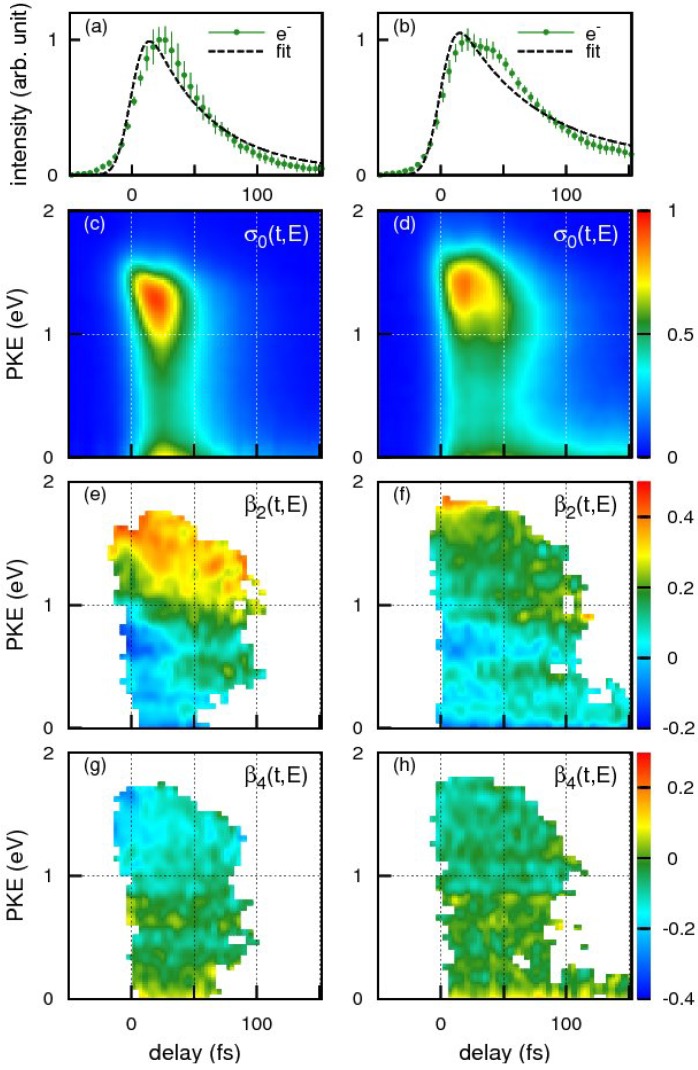
The time profiles of the photoionization signal intensity for (**a**) benzene and (**b**) toluene. Photoelectron signals are indicated by green dots with error bars. Time-energy maps of the photoelectron intensity, *σ*(*t*,*E*), for (**c**) benzene and (**d**) toluene. Time-energy maps of the photoelectron angular anisotropies, *β*_2_(*t*,*E*), for (**e**) benzene and (**f**) toluene and *β*_4_(*t*,*E*) for (**g**) benzene and (**h**) toluene. Data points for *β*_2_ and *β*_4_ with standard deviations smaller than 0.2 are shown (see text). Reproduced with permission from Ref. [72].

Figure 15a,b shows the time profiles of the photoionization signal intensity observed for benzene and toluene using the 198 nm pump pulse and 264 nm probe pulse, respectively. The broken lines are the best-fit single exponential decay function convoluted with the cross-correlation of the pump and probe pulses. The effective lifetime estimated from the fit for benzene and toluene were 48 ± 4 and 62 ± 4 fs respectively. Close examination of our experimental results, however, reveals that a single exponential decay model does not adequately reproduce the observed time profiles in either case. The observed non-exponential profiles are, in fact, reproduced better by assuming molecular response functions considering propagation of a wavepacket from the Franck-Condon region to the seam of crossings during which time no population decay occurs from *S*_2_. The arrival times of the wave packets at the seam of crossings are estimated as 33 fs for benzene and 41 fs for toluene: the subsequent decay time is 32 fs for benzene and 43 fs for toluene.

The photoelectron kinetic energy distributions at each time delay, *σ*(*t*,*E*), are extracted as shown in Figure 15c,d for benzene and toluene, respectively. The distributions consist of the *S*_2_ and *S*_1_ components that are largely different from each other. The *S*_2_ component mainly appears in 0–1.5 eV, while *S*_1_ < 0.3 eV: the difference of kinetic energies originate from the fact that the electronic energy difference between *S*_2_ and *S*_1_ is transformed into the vibrational energy in *S*_1_, and the vibrational energy is approximately conserved upon ionization [70,71,72]. Since the probe photon energy of 264 nm is insufficient to cover the entire Franck-Condon envelope in ionization from *S*_1_, the quantum yield of *S*_2_–*S*_1_ internal conversion cannot be evaluated accurately from our result.

**Figure 16:**
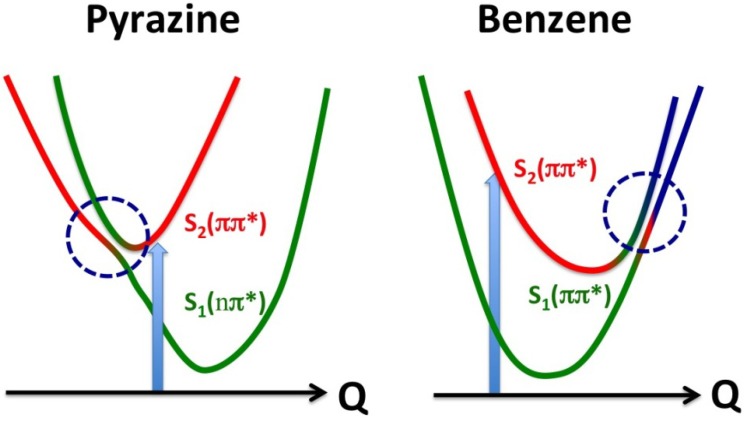
Comparison of conical intersections in pyrazine and benzene. The vertical arrows indicate photoexcitation, and the horizontal axes are the reaction coordinates. The red and green correspond respectively to the diabatic *S*_2_ and *S*_1_ states, and blue indicates contribution of other zero-order electronic states.

For both benzene and toluene, close examination of the high-energy (1–1.5 eV) region of the *S*_2_ component reveals vibrational wave packet dynamics. The molecules are planar in the Franck-Condon region of *S*_2_, and therefore ionization at *t* = 0 occurs to low vibrational levels of the cation, which also has a planar structure. As the vibrational wave packet moves out from the Franck-Condon region, ionization starts occurring to vibrationally excited states of the cation, lowering the entire distribution in energy from the moment the photoelectron signal initially appears. Another interesting feature is an oscillatory component that appears as a red triangular shape in Figure 15c and a red-yellow region in Figure 15d. Close examination of Figure 15e for benzene reveals that *β*_2_ varies with time, most clearly around 0.7 eV; *β*_2_ is negative at *t* = 0 and gradually increases with time to be positive around ca. 30 fs. Similar time dependence of *β*_2_ is also seen in Figure 15f for toluene, for example at around 1.0 eV. These rapid changes of *β*_2_(*t*,*E*) with time indicate variation of the electronic character along the out-of-plane distortion. When benzene undergoes out-of-plane distortion, it loses the planarity and, consequently, distinction between the *σ* and *π* electrons. Thus, the electronic characters of the *S*_2_ and *S*_1_ states gradually change along the reaction pathway. This is contrasted with the case of pyrazine, which has a conical intersection near the minimum of the diabatic *S*_2_ surface and the subsequent dynamics primarily occur in the planar geometry (Figure 16).

## 7. Summary

Ultrafast internal conversion via conical intersections plays a crucial role in the photophysics and photochemistry of aromatic molecules. These processes can be studied in the time and frequency domains by various types of photoelectron spectroscopies. Although theoretical studies have been performed on the *S*_2_–*S*_1_ internal conversion in pyrazine as a benchmark system, its real-time observation was only enabled by the development of sub-20 fs ultrafast lasers operating in the deep UV region. The most useful observable for studying the nonadiabatic electronic dynamics of pyrazine is the time–energy map of photoelectron anisotropy. On the other hand, the observation region of the excited state surfaces depends on the probe laser wavelength. We expanded the observation region using a vacuum ultraviolet free electron laser; however, fluctuation of the laser pulses due to the principle of self-amplification of the spontaneous emission restricted the time resolution. Filamentation four-wave mixing successfully generates sub-20 fs 159 nm pulse at 1 kHz, which allowed vacuum ultraviolet time-resolved photoelectron imaging with an ultimate time-resolution of 17 fs. A conical intersection similar to the *S*_2_–*S*_1_ system is observed for the cation states of *D*_1_ and *D*_0_. Pulsed field ionization photoelectron spectroscopy is affected by the strong vibronic coupling mediated by the conical intersection and its spectral feature for *D*_1_ differs strikingly from that in He(I) photoelectron spectroscopy. Because the molecular Rydberg states have essentially the same electronic potentials as the cation states, conical intersections occur between the Rydberg states with the *n*^−1^ and *π*^−1^ ion cores. Benzene and toluene have the *S*_2_–*S*_1_ conical intersection at non-planar geometries. When these molecules are photoexcited to *S*_2_, the nuclear wave packet travels more than 50 fs to reach the conical intersection region at non-planar geometry, and they undergo internal conversion. Along the reaction pathway, the loss of the planarity induces gradual change of the electronic character, which is manifested by time-evolution of the photoelectron anisotropy parameter.
